# Collagen Type I Improves the Differentiation of Human Embryonic Stem Cells towards Definitive Endoderm

**DOI:** 10.1371/journal.pone.0145389

**Published:** 2015-12-29

**Authors:** Camilla Holzmann Rasmussen, Dorthe Roenn Petersen, Jonas Bech Moeller, Mattias Hansson, Martin Dufva

**Affiliations:** 1 Novo Nordisk A/S, Maaloev, Denmark; 2 DTU Nanotech, Technical University of Denmark, Kgs. Lyngby, Denmark; University of Newcastle upon Tyne, UNITED KINGDOM

## Abstract

Human embryonic stem cells have the ability to generate all cell types in the body and can potentially provide an unlimited source of cells for cell replacement therapy to treat degenerative diseases such as diabetes. Current differentiation protocols of human embryonic stem cells towards insulin producing beta cells focus on soluble molecules whereas the impact of cell-matrix interactions has been mainly unattended. In this study almost 500 different extracellular matrix protein combinations were screened to systemically identify extracellular matrix proteins that influence differentiation of human embryonic stem cells to the definitive endoderm lineage. The percentage of definitive endoderm cells after differentiation on collagen I and fibronectin was >85% and 65%, respectively. The cells on collagen I substrates displayed different morphology and gene expression during differentiation as assessed by time lapse studies compared to cells on the other tested substrates. Global gene expression analysis showed that cells differentiated on collagen I were largely similar to cells on fibronectin after completed differentiation. Collectively, the data suggest that collagen I induces a more rapid and consistent differentiation of stem cells to definitive endoderm. The results shed light on the importance of extracellular matrix proteins for differentiation and also points to a cost effective and easy method to improve differentiation.

## Introduction

Human embryonic stem (hES) cells are characterized by the ability to self-renew and differentiate into mature cell types [1]. hES cells therefore hold the potential to be an unlimited cell source to generate differentiated cells for studying human development and for cell replacement therapy to treat degenerative diseases, including diabetes mellitus, Alzheimer’s disease and heart diseases [1–3].

hES cells are differentiated into mature cell lineages following a stepwise protocol, mimicking the *in vivo* development of the desired cell type or organ. Generation of definitive endoderm (DE), one of the three primary germ layers in the early embryo, is the first differentiation step for multiple cell lineages with significant interest for cell replacement therapy, including pancreas, liver, gut and lung [2,4]. Several studies have developed differentiation protocols of hES cells towards DE with soluble growth factors and small molecules targeting selected signalling pathways, including Wnt, TGFβ and AKT/PI3 [5–8].

The potential clinical applications of hES cells are hampered by the lack of efficient fully defined and xeno-free differentiation protocols that gives rise to the functional, mature cell type of interest [9]. The majority of stem cell research focuses on the effect of growth factors and their downstream signalling pathways’ role in differentiation, whereas the physical microenvironment including the extracellular matrix (ECM) composition has been left mainly unattended. However, accumulating evidence demonstrates that the ECM greatly influences cellular behaviour such as cell differentiation [10]. Several cell receptors specific for ECM proteins (ECMPs) have been identified, such as the heterodimeric intregrins [11,12]. Besides providing adherence for cells, the integrin-ECM interaction provides bidirectional signalling, which serve as a link between the intracellular and extracellular environment and activates downstream signalling pathways. These downstream signalling pathways include MEK-ERK and PI3-kinase, which are involved in regulating self-renewal, differentiation and migration in hES cells [11,12].

Several studies have demonstrated that the ECM has an essential role in embryonic development, both in the early state [13–15] and later in the organogenesis [16,17]. Furthermore, ECMPs and their corresponding integrins play a role in self-renewal, proliferation, differentiation of mouse and human embryonic stem cells *in vitro* [18–22]. Current coating substrates for culturing ES cells, such as Matrigel (BD Bioscience) or single ECMPs, like fibronectin (Fn), greatly support cell adhesion, but they do often not mimic the extracellular environment the cells are exposed to *in vivo*. Here we employed microarrays spotted with combinations of ECMPs to systematically identify ECMP combinations that induce hES cell differentiation into DE. The ECMP microarray screen found several ECMP candidates that enhance DE cell yield and purity.

## Materials and Methods

### Cell culture and differentiation

The cells were cultured at 37°C in a humidified incubator with 5% CO_2_. The hES cells (SA121, Takara Bio Europe AB) were cultured in the DEF-CS™ 500 system (Takara Bio Europe AB). For differentiation, the cells were dissociated into single cell suspension with TrypLE Select (Invitrogen) and seeded at 40.000cells/cm^2^ in DEF-CS medium according to the supplier’s instructions [23]. The cells were kept undifferentiated in DEF medium for 3 days. Differentiation to DE cells was done in basal medium containing, RPMI1640 + Glutamax (RPMI) medium (Gibco) with 2% B27 without insulin (Gibco) and 0.1% Pen strep (Invitrogen). On day 4 after cell seeding, the cells were rinsed with basal media and basal media with 100ng/ml recombinant mouse Wnta3 (R&D Systems) was added. On day 5 after cell seeding, the cells were rinsed with basal media and basal media with 10ng/ml Activin A was added (Peprotech). Fresh basal media with 10ng/ml Activin A was added on day 6 and 7. Cells from different passages were used as biological replicates. Viability and number of cells before plating were measured using a NucleoCounter® NC-3000TM (Chemometec, DK) according to manufacturer’s instructions.

### Preparation of extracellular matrix coated substrates

The ECMP microarray slides were prepared with a non-contact micro-dispensing robot (Nanoplotter^TM^ (GeSiM, Germany)). The Nanoplotter had cooling of the microtitre plate and the humidity was kept to 60% during the spotting to avoid rapid evaporation. Solutions of ECMP and PBS (Gibco) were mixed in 384 microtitre plates. 487 combinations of ECMPs were made from 14 ECM proteins and peptides, which were selected from literature studies and represent the major ECM families. The following ECMPs were used: Fibronectin (Sigma) (Fn), laminin 511 (Biolamina) (Ln511), laminin 111 (Biolamina) (Ln111), laminin 521 (Biolamina) (Ln521), laminin from human fibroblast (Sigma) (LnH), vitronectin (Sigma) (Vn), fibronectin adhesions promoting peptide (Sigma) (FnAd), collagen I from rat tail (Gibco) (Col1), collagen IV (R&D Systems) (Col4), collagen II (Millipore) (Col2), collagen III (Millipore) (Col3), collagen V (Millipore) (Col5), heperan sulphate proteoglycan (Sigma) (Hep), nidogen 1 (R&D Systems) (Nid) and netrin 1 (R&D Systems) (Ne). The concentrations of the individual ECMP reflect the recommendations from of the suppliers. Further details about the ECMPs are described in S1 Table. 10% (w/v) bovine serum albumin (BSA, Sigma) was used as a negative control because it is an unrelated protein to ECMP. Each protein combination (S1 Table) was spotted in four replicates per array using a Nano-tipA (GeSiM) with the following tip settings: 150 droplets per spot, 250μs delay, 50μs pulse, 75 V, 100hz and 1.1mm distance between each spot. The resulting spot diameter was about 400±50μm. The 487 protein combinations and replication required two microscope slides. The slides were made of polystyrene (Nunc) the same material as cell culture dishes. The ECMP microarray slides were stored at -18°C until use, with a maximum storage time of 3 weeks. The slides were transferred to cell culture dishes fitted to microscope slides (Nunc, Denmark) and they were incubated at 37°C for two hours to ensure complete absorption of ECMPs. Subsequently, the slides were sterilized with 70% ethanol for 30 minutes and washed 3 times with PBS and were incubated at 37°C until seeding of the cells. Microtitre well plates (Cellbind, Corning) were coated by adding ECMPs diluted in PBS and incubated at 37°C for 1–2 hours. The ECMP dilution was removed immediately before seeding of cells, leaving the well plate constantly wet.

### Immunofluorescence staining and microscopy

The cells were fixed with 4% formaldehyde (Lilly’s fixative, Mallinsckridt Baker) for 20 minutes at room temperature. The samples were permeabilized with 0.5% Triton-X-100 (Sigma Aldrich) in PBS for 10 minutes at room temperature and subsequently blocked for 30 minutes using TNB blocking buffer (0.1M tris-HCL pH 7.5, 0.15M NaCl and 0.5% blocking reagent from Perkin Elmer TSA kit). Primary antibodies were diluted in 0.1% TritronX-100 in PBS and applied to the samples followed by incubation overnight at 4°C. The following primary antibodies were used: goat polyclonal anti Sox17 (1:1000) (R&D Systems, AF1924), mouse polyclonal anti Oct3/4 (1:500) (Santa Cruz, sc5279). The samples were washed three times with PBS and the secondary antibodies Alexa Fluor 594 conjugated donkey-anti-mouse IgG (Invitrogen) and Alexa Fluor 488 donkey-anti-goat IgG (Invitrogen) were added in a 1:1000 dilution together with DAPI (1:2000) (Sigma- Aldrich) and occasionally Alexa Fluor 488 Phalloidin (1:20) (Invitrogen) in 1% TritronX-100 in PBS for 45 minutes at room temperature. The samples were rinsed three times with PBS for five minutes.

Microscopy images were obtained on the InCell analyzer 2000 (GE Healthcare). A 10X/0.45 Plan Apo objective was used with laser auto focus. For the microarray screen one image per spot was taken, whereas in well plate formats 9–16 images per well were taken. Images were analysed and quantified using the InCell Developer Toolbox 1.9.2 (GE healthcare). Representative images were processed in ImageJ or InCell Developer Toolbox. Example images are given in S2 Fig.

### Quantitative Polymerase Chain Reaction (qPCR)

RNA was isolated and purified from cell cultures using Nucleospin RNA Kit (Macherey-Nagel) and RNA concentration and purity were measured with the NanoDrop ND-100 spectrophotometer (Thermo Scientific). cDNA was prepared from 500ng RNA per sample using the iSCript cDNA Synthesis kit (Bio-rad) and the cDNA was subsequently diluted 10 times. qPCR was performed using 1/100 of the cDNA, 500nM each of the respective forward and reverse primers and 2x Brilliant III Ultra-FastSYBR Green master mix (Agilent) in 10μl reactions. Primer sequences are listed in S2 Table The reactions were performed on a MX3005P qPCR system (Agilent) using the following program; 95°C for 3 minutes, 40 cycles of 95°C for 15 seconds 60°C for 20 seconds, followed by a melting curve cycle of 95°C for 20 seconds and 55°C for 30 seconds. Three independent biological replicates were obtained with hES cells from different cell passages. For each biological replicate, technical triplicates were analysed. The two housekeeping genes *TBP* and *GUSB*, were used to normalize the data [5].

### Statistical Analysis

For the ECMP micro array screen four biological replicates with hES cells from different cell passage where used and each biological replicate contained four technical replicas for each ECMP combination. Data from the ECMP microarray screen was normalized by calculating a z-score for each spot:

$$Z_{\mathrm{Sox}17}\;=\;\frac{\left(R_{\mathrm{Sox}17}-\mu_{\mathrm{Sox}17}\right)}{\sigma_{\mathrm{Sox}17}}$$

R_iSox17_ was the ratio between the number of Sox17 positive cells and total cells (DAPI stained) for the given spot. μ_Sox17_ was the average of Sox17/DAPI ratio of all the spots in the given experiment (N1-N4), whereas σ_Sox17_ was the standard deviation of Sox17/DAPI ratio of all the spots in the given experiment. In addition a z-score for number of cells (DAPI) was calculated in a similar way:

$$Z_{\mathrm{DAPI}}\;=\;\frac{\left(R_{\mathrm{DAPI}}-\mu_{\mathrm{DAPI}}\right)}{\sigma_{\mathrm{DAPI}}}$$

where DAPI was the number of DAPI positive cells. Z-scores from replication spots (four per ECMP combination) were averaged, and the given values were an average from the four independent experiments (N1-N4).

For the experiments in the microtitre plates, paired–test was used for statistical analysis and values in graphs were presented as mean ±S.E.M of at 3–5 independent biological replicas with hES cells from different cell passage.

### Gene expression analysis using DNA microarrays

RNA was isolated as described above and the quality of RNA samples was measured using the 2100 Bioanalyzer (Agilent) and only samples with RNA integrity number (RIN) above 7 were used. Microarray analysis was performed by the array facility DMAC at Technical University of Denmark. RNA samples were prepared according to the protocol One-Color Microarray-Based Gene Expression Analysis, Low Input Quick Amp Labelling, Version 6.6, September 2012 (Agilent Technologies, USA). 100ng total RNA per samples was used. Samples were subsequently hybridized to Agilent SurePrint Whole Human Genome (4x44K) Oligo Microarrays and scanned as described by the manufacturer (Agilent Technologies, Santa Clara, CA, US) using the DNA microarray scanner from Agilent Technologies. The raw fluorescent data was extracted using the Agilent Feature extraction software.

Batch effects were removed using the ComBat function of the sva R package for modelling and intensity values were normalized between expression arrays by applying quantile normalization [24]. t-test using the eBayes function of the limma R package was used to test significance of differential expression. Bonferroni correction and Benjamini-Hochberg correction were used to correct for multiple testing. The gene expression from cells cultured on the respective ECMP was compared to the gene expression of cells cultured on Fn. Pathway analysis was done using WikiPathways [25–28] using differentially expressed genes at the P<0.01 after Benjamini-Hochberg correction.

## Results

### Identification of extracellular matrix proteins combinations that promote definitive endoderm differentiation

The aim of this work was to identify ECMPs that promote the differentiation of hES cells into DE cells. hES cell to DE differentiation reach >90% on Fn using optimal protocols [29]. It is difficult to quantify possible positive effects of ECMPs with such high differentiation efficiency. Therefore a suboptimal DE differentiation protocol was evaluated. This protocol includes 100ng/mL Wnt3a and 10ng/mL Activin A and generates around 60% Sox17 (DE marker) positive cells when differentiated on Fn substrates (S1 Fig). Increasing the Activin A concentration to 100ng/mL resulted in >80% DE cells (S1 Fig) indicating that adding cues to the suboptimal protocol increased the DE differentiation. The suboptimal protocol was used to screen for ECMPs improving DE differentiation. The ECMP microarray screening method was developed and optimized, including selection of microscope slide material as well as selection of the size of the individual protein spots and the distance between them. Polystyrene (same material as tissue culture flasks) was superior to all other tested slide types. Differentiation of hES cells on Fn spotted polystyrene slides showed that 400±50μm spots, spaced 700 μm apart gave about 60% differentiation and thus similar as in microtitre plates.

**Fig 1:**
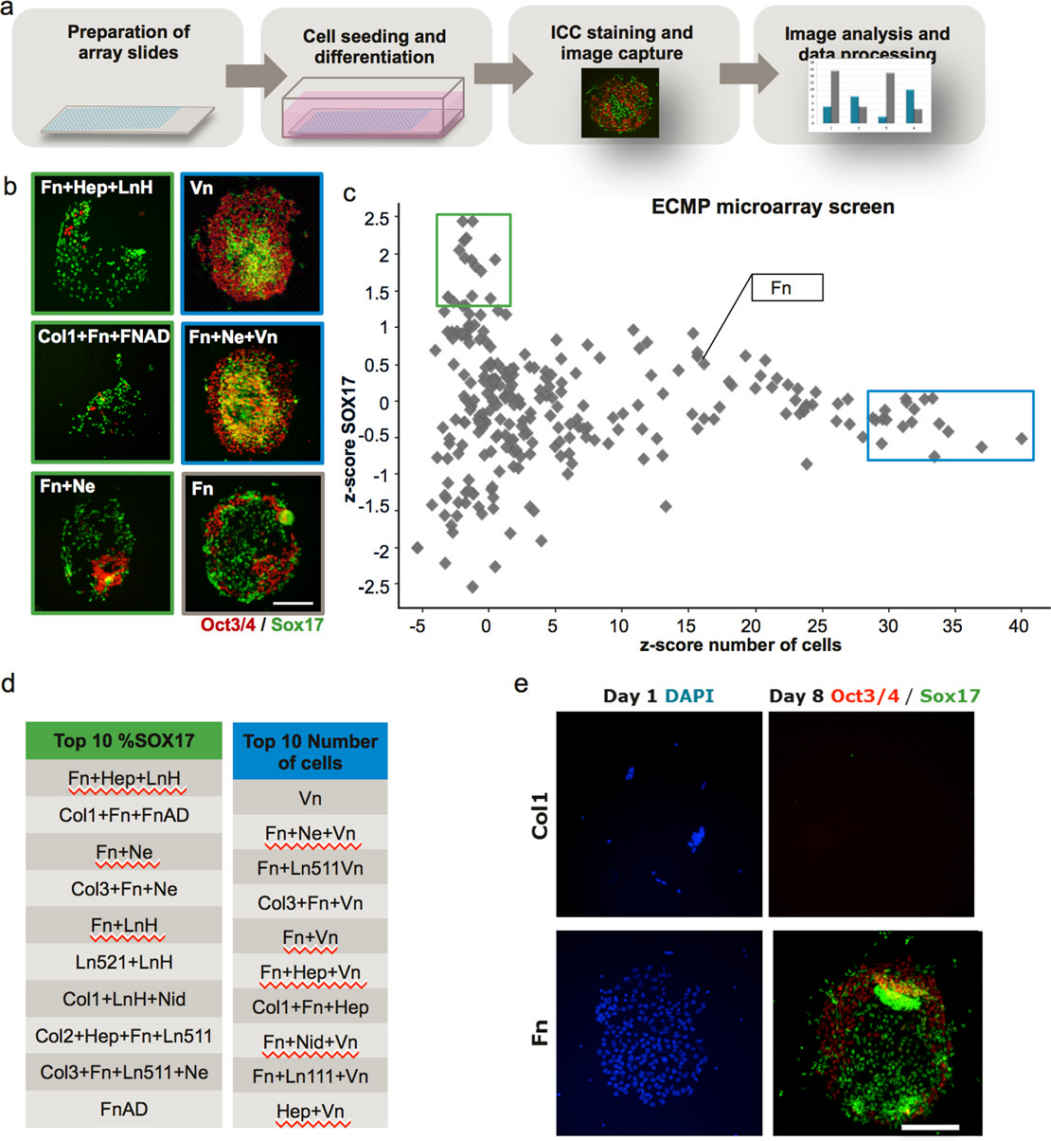
The microarray screen with combinations of ECMPs demonstrated that the DE differentiation was affected by the ECMPs. (**a**) Schematic overview of the ECMP microarray screen. The ECMP microarray slides were prepared by inkjet spotting and undifferentiated hES cells were subsequently seeded and differentiated towards DE. To assess the differentiation, the samples for immunofluorescence were stained for the markers Oct3/4 (undifferentiated hES cells), Sox17 (DE) and DNA (DAPI), and microscope images were captured using an automated microscope, InCell Analyzer. The images were quantified and the data was analysed. **(b**) Fluorescence microscope images of selected spots stained for Oct3/4 (red) and Sox17 (green) (scale bar = 200μm). (**c**) Sox17 z-scores plotted against cell number z-scores for each respective ECMP combination (n = 4). The green square indicate the ECMP combinations giving highest fraction of DE cells, whereas the blue square represents the ECMP combinations giving highest cell number. (**d**) Tables of the ECMP combinations giving to the 10 highest fractions of Sox17 positive cells and top 10 highest cell count respectively. (**e**) Immunofluorescence staining of spots from the protein microarray screen. Images were captured at day 1 (one day after seeding) and day 8 (at the end of the DE differentiation protocol) (Scale bar = 200μm).

A microarray of 487 different combinations of ECMPs (S1 Table) was used to screen for ECMP combinations that promote DE differentiation. DAPI staining of cells at day one after plating on the array showed that some ECMPs immobilized hES cells homogeneously and efficiently (e.g. Fn) while other ECMPs did not capture hES cells efficiently (e.g. pure Col1, Fig 1E). The hES cells did not attach to the negative control (BSA) spots. The differentiation was evaluated by quantitative immunofluorescence microscopy of Oct3/4 (pluripotent marker) and Sox17 (DE marker) followed by microscopy and image analysis. Examples of immunostainings of spots after differentiation are given in Fig 1B. Morphologically, cells on spots after differentiation often appeared in distinct clusters of Oct3/4 positive and Sox17 positive cells respectively. Large regions with 100% Sox17 positive cells were observed on the spots with Fn+Hep+LnH, Col1+Fn+FnAD and Fn+Ne. Also Oct3/4 positive cells occasionally displayed a sub-spot organization. Oct3/4 positive cells were organized in the periphery of the spots on some ECMP combinations (e.g. Fn+Ne+Vn and Vn alone, Fig 1B) or organized in scattered islands (e.g. on Fn+Ne). Some ECMPs spots, including pure Col1, contained too few or no cells to evaluate DE induction after differentiation (Fig 1E).

The numbers of Oct3/4 positive, Sox17 positive and DAPI positive cells on each spot were quantified and z-scores were calculated (Fig 1C). It was possible to identify ECMP combinations that produced a higher percentage of DE cells (Fig 1C, green box and Fig 1D) or the same percentage of DE cells but with higher cell counts (Fig 1C, blue box and Fig 1D) compared to cells on Fn. However, it was not possible to identify ECMPs that both resulted in high cell number and high DE differentiation fraction using the microarray screen. 9 of the top 10 ECMP combinations giving high Sox17 fraction were a mixture of ECMPs: Fn, Ne, Col1, LnH, Col2, Col3 and Hep in combinations seem to promote DE induction in the screen. The single most dominant ECMP resulting in high cell counts was Vn.

### Extracellular matrix proteins component analysis

**Fig 2:**
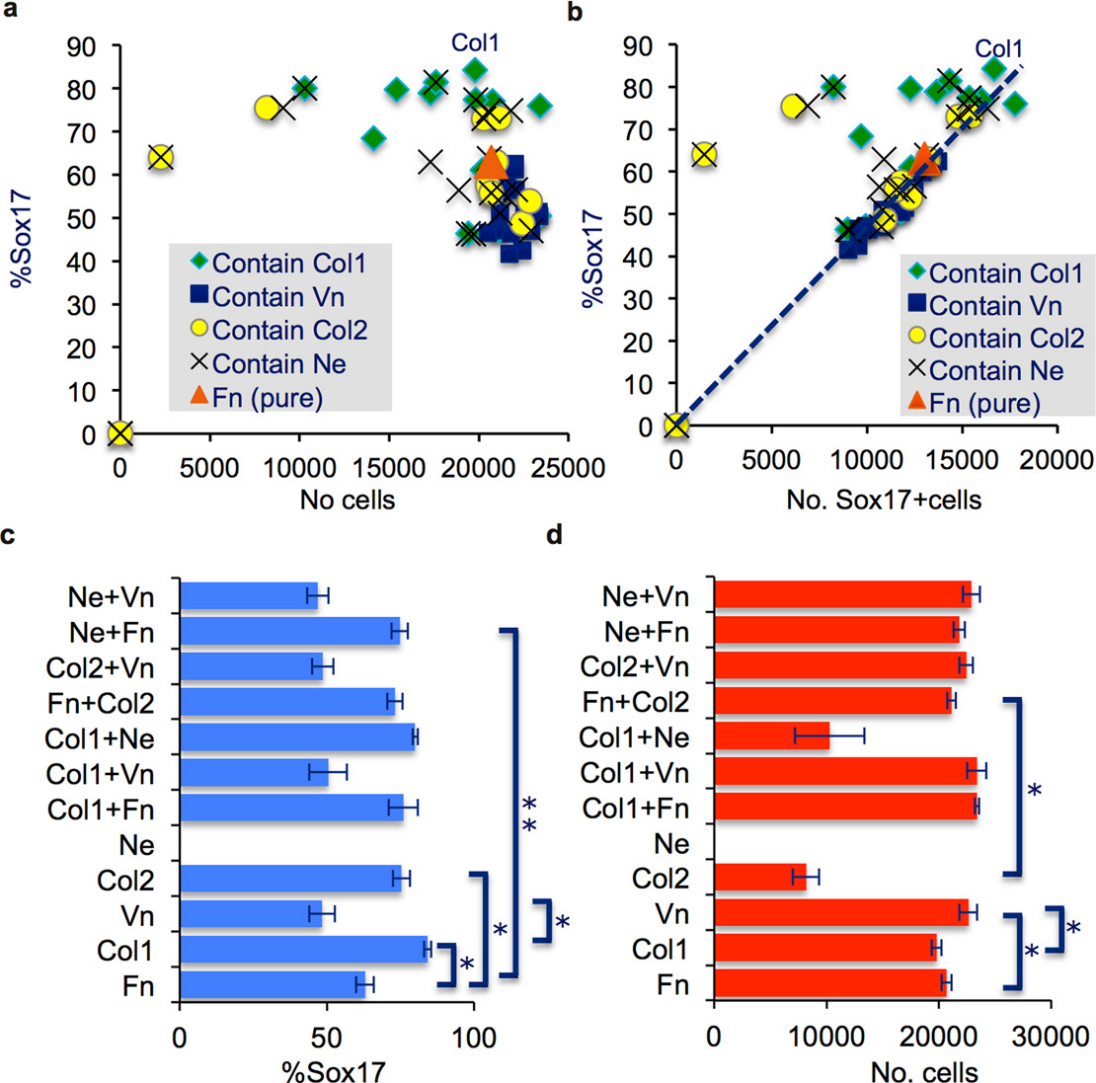
(a-d) DE differentiation of hES cells in microtitre plates on selected ECMP combinations. (**a-d**) Quantification of Sox17 positive cells and total cell number based on immunofluorescence image analysis of cells differentiated on different ECMP combinations in microtitre plates. n = 3–6, mean ± S.E.M.). * and ** indicate statistical significant differences to Fn, P<0.05 and P<0.005 respectively. Note that not all statistical significant differences are displayed in the graphs. Full data set is given in S3 Table. Abbreviations are explained in S1 Table. **(a)** Scatter plot of percentage of Sox17 positive cells and the total number of cells for each respective ECMP combination tested (see S3 Table for descriptions), **(b)** Scatterplot of percentage Sox17 positive cells and the total yield of Sox17 positive cells (calculated as percentage of Sox17 positive cells * total cell number /100). Linear regression of data excluding six outliers (out of 65) not considered following the linear correlation (see results). r^2^ = >0.95. **(c)** Percentage of Sox17 positive cells of a selected ECMP combinations. **(d)** The total cell number of a selected ECMP combinations.

The top 10 combinations based on z-scores (Fig 1C and 1D) from the ECMP microarray screen and new ECMPs combinations based on the top 10 combinations were further tested in corresponding microtitre plate assays, where the ECMPs were immobilized by 1-2h incubation in a buffer. Overall, the majority of the tested combinations supported good cell growth and/or cell attachment well as the majority of the ECMP combinations resulted in cell numbers >18000 (Fig 2A). All the ECMP combinations with high cell number during the differentiation in the ECMP microarray screen were also verified to have high cell number in the well plate format (S3 Table). Furthermore, these ECMP combinations only induced intermediate percentage of Sox17 positive cells (43–61%, Fig 2A, S3 Table), which confirm the data from the ECMP microarray screen. It appeared that Vn had a positive effect on the cell number in the well plate format (Fig 2A blue squares and S3 Table) but suppressed DE induction.

Seven of the top 10 ECMP combinations improving DE induction in the ECMP microarray screen were confirmed to improve DE induction in the microtitre plate format (Fig 1D, S3 Table). In general there was a strong correlation of the presence of Col1 in mixtures and high DE induction (Fig 2A green rhombs). The highest percentage of Sox17 positive cells (84%) was obtained with pure Col1 (Fig 2A and 2C). When Vn was present in ECMP combinations containing Col1, low DE induction was observed (47–56% Sox17 positive cells), (Fig 2A). This indicates the Vn has a suppressive effect on DE induction that dominates the positive effect of Col1 (Fig 2A and 2D). Vn had by contrast a positive effect on Col1’s ability to yield high cell numbers (Fig 2D). The presence of Col2 also correlated with high DE induction but less so than Col1 (Fig 2A). However, Col2 needed Fn as cofactor in order to also yield high cell numbers (Fig 2C and 2D). Ne contributed to DE induction, but only in combination with Fn (Fn+Ne, Fig 2C and 2D). Otherwise Ne together with other ECMP did not seem to have an additive effect on DE induction. Ne was associated with lower cell number yields after differentiation; Ne alone gave no cells and Col1+Ne had significantly reduced cell number compared to Col1 alone. By contrast, Fn seems to rescue poor cell yield of Ne and the combination resulted in high Sox17 induction and high cell yield (Fig 2C and 2D).

The total Sox17 positive cell yield ((percentage Sox17 positive * cell number)/100) was plotted (Fig 2B). The majority (59 out of 65) of the combinations followed a straight line (r^2^>0.95) suggesting that the Sox17 positive cell yield is a function of DE induction. I.e., the higher the DE induction frequency, the higher the Sox17 positive cell yields. However, this correlation was not seen in ECMP combinations containing Col1 and Col2, which had low cell yields while still giving high percentage of Sox17 positive cells (Fig 2B). The highest Sox17 positive cell yields were obtained using Col1+Fn but this combination resulted in a slightly lower Sox17 positive percentage (Fig 2C and 2D). The higher Sox17 positive yield was mainly due to higher total cell number of Col1+Fn compared to Col1 alone (Fig 2C and 2D).

### Kinetics of differentiation on different extracellular matrix proteins

**Fig 3:**
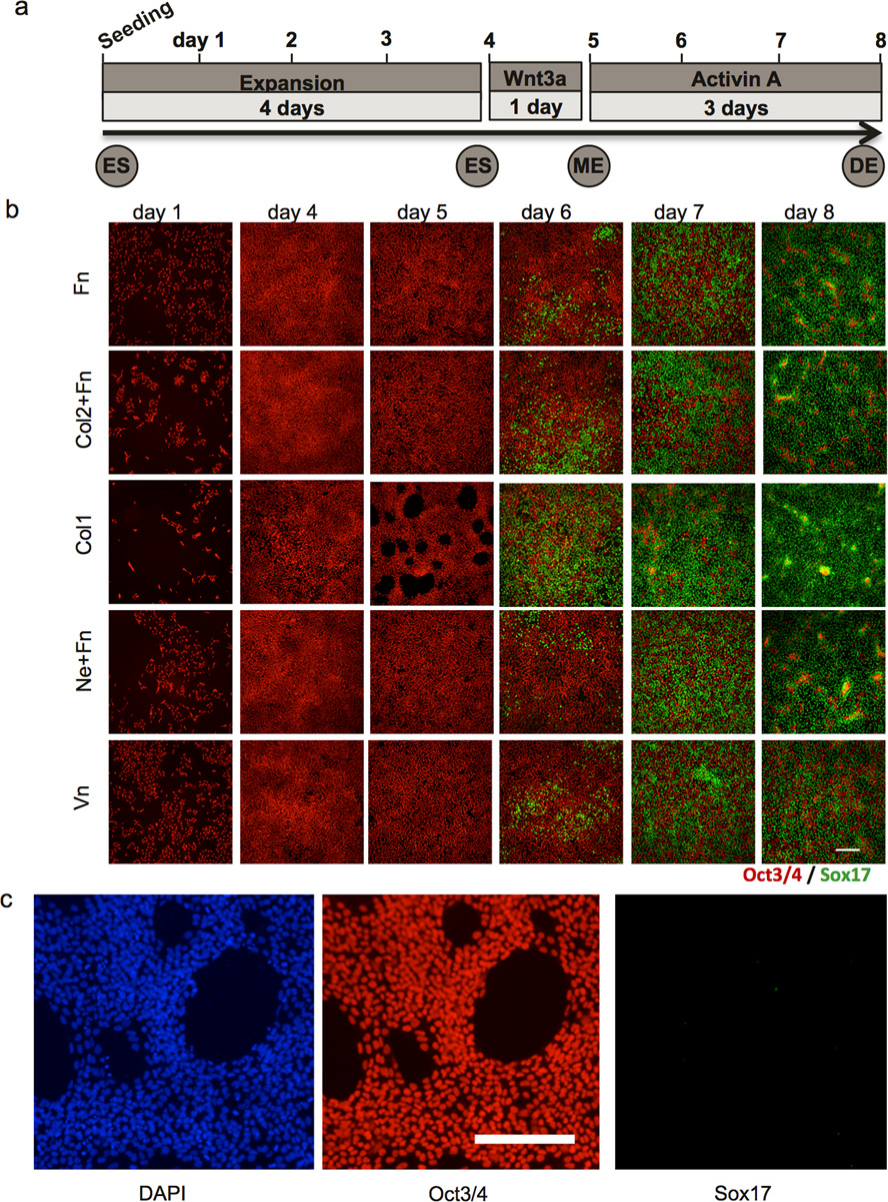
The dynamic differentiation of hES cells to DE is influenced by ECMPs. (**a)** Schematic overview of the semi-optimal protocol for DE differentiation. hES cells were seeded and kept undifferentiated for 4 days to allow expansion. Subsequently the cells were treated with Wnt3a to direct the cells from the pluripotent stage into the mesendoderm stage, followed by 3 days with Activin A to direct the cells from the mesendoderm stage to the DE stage. **(b)** Representative immunofluorescence images from three independent experiments of cells cultured on the five different ECMP combinations at different time points during the DE differentiation protocol. The tested ECMP combinations were fibronectin (Fn), collagen II+fibronectin (Col2+Fn), collagen I (Col1), netrin 1+fibronectin (Ne+Fn) and vitronectin (Vn) (scale bar = 200μm). **(c)** Magnification of cultures on Col1 at day 5 (scale bar = 200μm).

**Fig 4:**
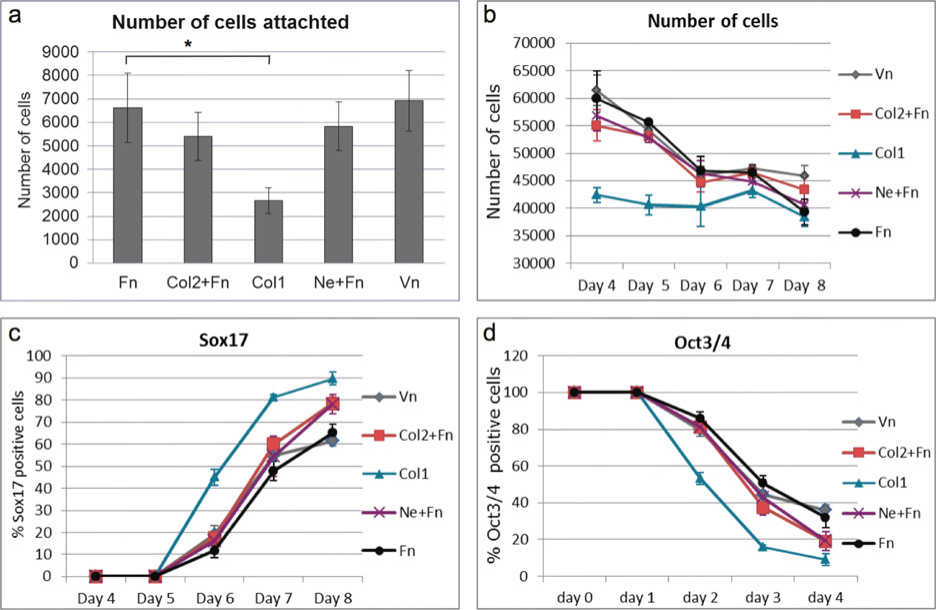
Quantitative immunofluorescence of cell cultures at different time points during the DE differentiation protocol on the different ECMP substrates. (**a**) Quantification of number of cells, which were attached 24h after seeding on the respective ECMP substrates (day 1). (**b**) Quantification of the number of cells at the different days during DE differentiation. (**c**) Quantification of the fraction Sox17 positive cells different days during DE differentiation. (**d**) Quantification of the fraction Oct3/4 positive cells at different days during DE differentiation. (n = 3, mean ± S.E.M. and * indicate statistical significant differences, P<0.05). ECMP abbreviations are explained in legend in Fig 3.

**Fig 5:**
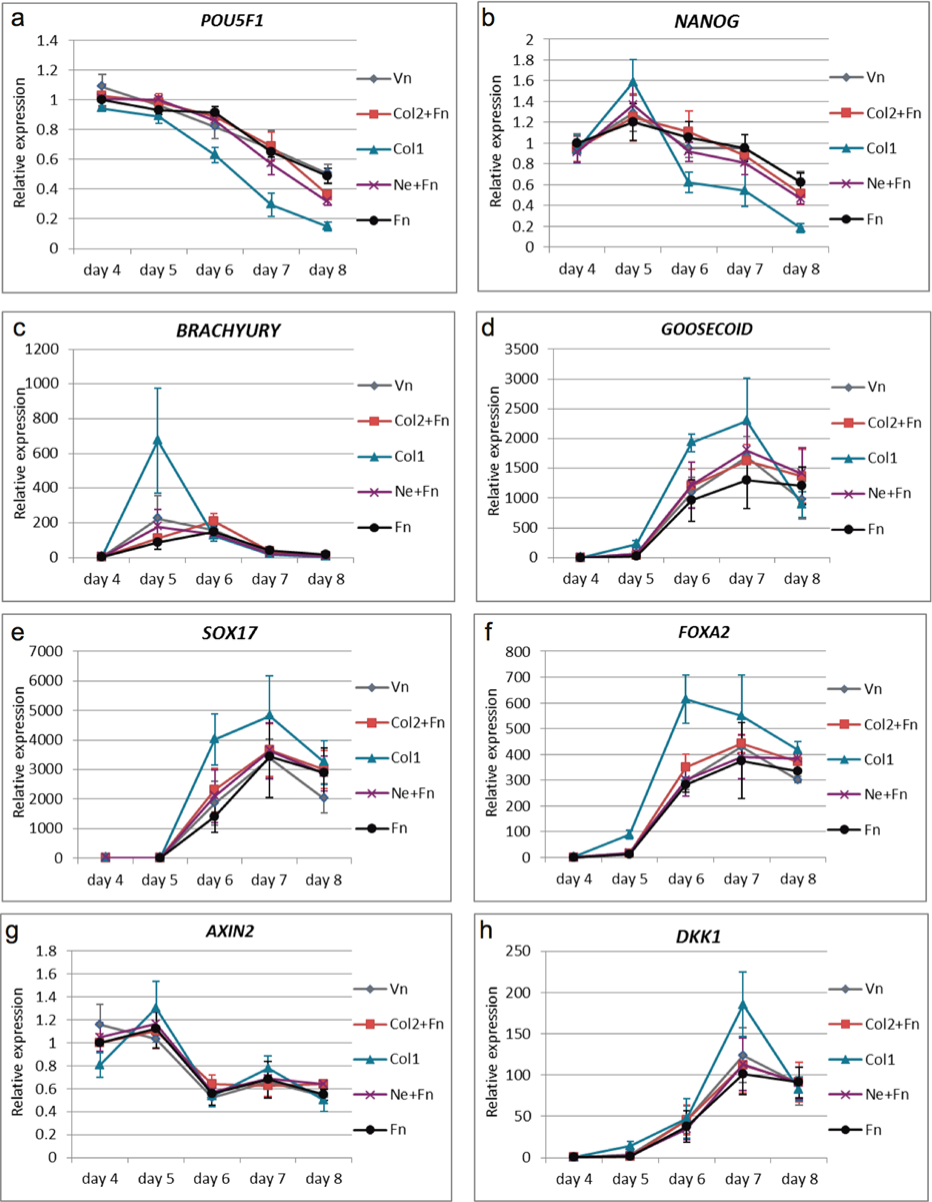
Gene expression analysis for markers at different time points during DE differentiation. Following genes were analysed; the pluripotent markers *POU5F1* (**a)** and *NANOG* **(b)**; mesendoderm/early DE marker *BRACHUYRY* **(c);** the DE markers *GOOSECIOD* **(d),** *SOX17* **(e),** *FOXA2* **(f);** the WNT downstream target *AXIN2* **(g);** and the WNT inhibitor *DKK1*. (n = 3, mean ± S.E.M.). Abbreviations are explained in legend Fig 3.

In order to pinpoint the time when the effect of different ECMPs affects differentiation, a time course study was performed. Five ECMP coatings were selected for the time course studies of DE differentiation. Col1, Col2+Fn and Ne+Fn were selected because their ability to induce DE and that they represent different types of ECMPs. Fn was used as a cofactor to Col2 and Ne respectively in these experiments. Vn was selected based on its poor ability to induce DE and to contrast the effects of the high performing ECMPs. Pure Fn was used as a reference. The cells were analysed at each time point by immunofluorescence (Figs 3 and 4) and quantitative PCR (Fig 5). hES cells initially attached (day 1) in tight clusters on Col1 and Col2+Fn coated wells. By contrast, the hES cells seeded on Fn and Vn were evenly distributed (Fig 3). The number of hES cells attached to Col1 coated wells one day after seeding (day 1) was significant lower than the number of cells on Fn coated wells (Fig 4A), corroborating the findings in the ECMP microarray screen (Fig 1E). The other investigated combinations resulted in similar number of cells attached as on Fn (Fig 4A). Three days in pluripotent culture conditions expanded the number of cells about 8–10 fold from initial attachment cell numbers (Figs 3, 4A and 4B respectively). However, the Col1 coating resulted in about 16-fold increase in cell number (Fig 4A and 4B). At this stage all the cell cultures were confluent. The total number of cells decreased throughout differentiation for all ECMPs except for Col1 where the total number of cells was stable (Fig 4B). At day 8, the total number of cells was at similar level for all ECMPs except for Vn, which had higher number of cells (Fig 4B). After one day with Wnt3a treatment (day 5), the overall culture morphology differed greatly in Col1 cultures compared to the other ECMPs. Cells adhered tightly together on Col1 and holes in the cell layer appeared (Fig 3C). It should be noted that the morphology of individual cells did not differ on the respective substrate (S3 Fig). Sox17 positive cells appeared on day 6 in all cultures (one day with Activin A). The Col1 coating had about 45% Sox17 positive cells on day 6 while the Fn coating had about 12% Sox17 positive cells. The cell culture morphology indicated that the holes formed at day 5 were repopulated within a day. It appeared that both Sox17 positive cells and Oct3/4 positive cells repopulated the holes in the cell layer observed at day 5 on Col1. The percentage of Sox17 positive cells increased for all the ECMPs during differentiation with Activin A (Figs 3 and 4). The Col1 coating had a higher percentage of Sox17 positive cells compared to Fn coating throughout the entire differentiation process (Fig 4C). Ne+Fn and Col2+Fn followed the differentiation pattern of Fn until the last day, day 8, where these ECMP combinations resulted in higher percentage of Sox17 positive cells compared to Fn (Fig 4C). Vn coated cultures followed Fn coated cultures in the percentage of Sox17 positive cells throughout the differentiation (Fig 4C). Sox17 positive cells had similar morphology on all substrate (S3 Fig).

To characterize the DE differentiation and to identify any potential differences between the ECMPs, the expression of different genes were assessed for the five time points (Fig 5). The expression of the pluripotent gene *POU5F1* (*OCT3/4*) [30] declined in general throughout the differentiation towards DE irrespective of ECMPs used. Cells on Col1 coating had a significant lower expression (P<0.05) of *POU5F1* compared to cells on Fn coating from day 6 and onwards (Fig 5A). The expression of the pluripotent marker *NANOG* [31] increased after Wnt3a priming (day 5) and subsequently declined throughout the differentiation (Fig 5B) which is in accordance with previous results [32]. At day 5, cells on Col1 and Ne+Fn coatings had significant higher (P<0.05) expression level of *NANOG* compared to cells on Fn coating. At day 8, cells on Col1 had significant lower (P<0.05) *NANOG* expression level compared to cells on all other ECMP coatings. Cells on Col2+Fn and Ne+Fn coatings followed cells on Fn coating in the relative expression of *NANOG* after day 4 (Fig 5B). The expression of *BRACHUYRY*, a transient mesendoderm marker [7,33], increased during the first days of differentiation and subsequently declined, which corroborates previous studies [5,32]. The expression of *BRACHUYRY* in cells on Col1 coating increased 675 fold on day 5 compared to day 1 (Fig 5C). The expression was significantly higher (P<0.0005) than in cells on Fn coating. The *BRACHUYRY* expression for cells on Ne+Fn and Vn coatings also peaked on day 5, where the expression was significantly higher than in cells on Fn coating (P<0.05) but not as high as cells on Col1. However, for cells on Fn and Col2+Fn coatings, the expression of *BRACHUYRY* peaked one day after the other ECMPs (day 6), and only with about a 200-fold increase in expression (Fig 5C). The expression of the anterior mesendoderm marker *GOOSECOID* [34], appeared 1–2 days later than *BRACHUYRY*, which is comparable to the results of previous studies [5]. The cells on the different ECMPs displayed comparable gene expression profiles for *GOOSECOID* and *SOX17*. These genes were upregulated on day 6 and 7 and decreased on day 8. There was no significant difference in *GOOSECOID* (Fig 5D) or *SOX17* (Fig 5E) expression in cells on the different ECMPs tested. However, there was a tendency that cells on Col1 had higher induction of these genes. *FOXA2*, a DE marker [35], had similar expression pattern as *SOX17* and *GOOSECOID*. However, the *FOXA2* expression for cells on Col1 coating peaked at day 6, whereas the expression of *FOXA2* on the other ECMPs peaked at day 7 (Fig 5). The expression of *AXIN2*, a downstream target of WNT signalling [36], were similar in cells on all the tested ECMPs. The expression increased on day 4 and subsequently declined (Fig 5G). In contrast, the expression of *DKK1*, a Wnt inhibitor [37], was significant higher on day 7 in cells on Col1 coating compared to cells on Fn coating (P<0.05). The *DKK1* expression in cells on the other ECMPs was similar to cells on Fn coating (Fig 5H). Quantitative PCR of *CXCR4* showed that all ECMPs induced *CXCR4* expression after the 8 day differentiation protocol (S4 Fig) indicating that the cells where differentiated towards definitive endoderm and not visceral endoderm [38]. To summarize, kinetics of the majority of the investigated genes were similar for the ECMPs and concur with previous studies of DE differentiation. However, the data suggests that cells on Col1 entered the differentiation path through earlier and stronger mesendoderm induction compared to cells on other substrates.

### Gene expression arrays

Global gene expression analysis was used to investigate the cell populations obtained after 8 days growth and differentiation in order to evaluate how similar the cells are after differentiation on different substrates. The data showed that only cells cultured on Col1 showed significant changes in gene expression compared to cells cultured and differentiated on Fn (the data is summarised in S4 and S5 Tables and completely presented in S6–S10 Tables). About 92 genes were differentially expressed in cells on Col1 compared to cells on Fn using Bonferroni correction (P<0.05). The results also showed that the qPCR data was confirmed by the DNA microarray analysis (Fig 5 and S4 Table). In general, there were only modest differences in gene expression profile of cell differentiated on Col1 compared to cells on Fn (S5 Table) indicating that the cells on the respective substrate were largely similar. The top downregulated gene (about three-fold) was *DACT1* which is an inhibitor of Wnt signalling [39,40] (S5 Table). Other downregulated genes were *POU5F1* (2.8-fold) and *SOX2* (2.5-fold), which are markers for pluripotent cells, indicating a better differentiation on Col1. One interesting gene that was upregulated was Nitric oxide synthase 2 (*NOS2*) (2.6-fold), which has been shown to activate beta-catenin and EMT in breast cancer cells [41]. It should be noted, that any transient effects of Col1 observed at day 5–6 (Fig 5) would not be expected to be detected by the DNA microarray analysis since it was only conducted for day 8. Pathway analysis of the differentially expressed genes showed that cells cultured on Col1 compared to cells cultured on Fn had differentially expressed genes involved in focal adhesion, neural crest differentiation, spinal cord injury and Notch signalling.

## Discussion

Several recent studies have highlighted the essential role of ECM in stem cell biology [19–21], and here we demonstrate that the ECMP composition influence the differentiation of hES cells towards DE. A semi-effective Wnt3a and Activin A based DE differentiation protocol was used in order to be able to observe any positive effects of the ECMPs. Col1, Col2 and Ne improved the efficiency of DE differentiation of hES cells, whereas Vn appears to have a repressive effect but promotes cell adhesion and/or proliferation. Col2 and Ne respectively required that Fn was present in order to get both high percentage of DE differentiation and high cell numbers. It is likely that Col2 improves differentiation while Fn ensures good cell adhesion. Similar functional division is likely for Ne+Fn as Ne alone does not support cell attachment and/or growth. However, all the analyses pointed to Col1 having unique properties compared to Fn and the other tested ECMP combinations. Cell cultures on Col1 had distinct morphology, the cells proliferated faster and importantly gave purer DE cultures with very few Oct3/4 positive cells (Figs 3 and 4), potentially through a stronger induction of mesendoderm as discussed below. Given the few genes (92 genes) that showed differential expression, the relatively small amplitude of differential gene expression (S5 Table), and that most of these differentially expressed genes are fully explained by different Sox17/Oct3/4 positive cell ratio (deduced from Figs 2 and 3) in the respective cultures, the cells differentiated for 8 days on Col1 are likely very similar to cells differentiated on Fn.

Col1 is the most abundant ECMP in the human body and is mainly expressed in the intrinsic part of the ECM. In general Col1 provides structural support and binds other ECM proteins [42,43]. To our knowledge, Col1 has not previously been linked to development of DE or the *in vitro* differentiation of hES cells towards DE. However, a previously published array study reported that Col1 had a negative effect on hES cells differentiation to DE [18]. Their experimental ECMP microarray screen setup resembles ours. However, our results suggested that Col1 spots on the arrays did not bind hES well (Fig 1E) providing an explanation to the negative results of Col1 on the array platform. It is unclear if Brafman et al. [18] tested differentiation of stem cells on Col1 in the microtitre plate format where we see a large positive effect of Col1. Another array study have demonstrated that Col1 had a positive effect on the differentiation of mouse ES cells towards hepatic linages [44]. Col1 is not expressed by mouse ES cells in various cell culture conditions [19] and *in vivo* disruptions of the Col1 is not lethal until a late stage of development, due to aortic rupture [45]. To our knowledge Col1 is not expressed in the embryonic basement membrane through which epiblast cells ingress during gastrulation [46], however it is unknown whether it is present in the early embryo or extra embryonic tissue.

The gene expression analysis using qPCR suggested that Col1 induced a more rapid and consistent mesendoderm differentiation (day 5), resulting in more than 4-fold larger fraction of Sox17 positive cells on day 6 compared to cultures on Fn. Col1 was not good support for attachment of cells (Fig 4A) but supported cell proliferation to relatively high cell densities prior to Wnt3a priming. It is unclear if Col1 cultures could reach higher densities if incubated with pluripotency medium for a longer time in order to compensate for the significantly fewer cells on Col1 directly after cell seeding (Fig 4A). The holes observed on Col1 cultures appeared the day after Wnt3a treatment and the holes were covered with cells after one day of Activin A treatment (Fig 3B). This relatively rapid appearance of cells and disappearance of the holes suggests active migration of cells or movement of the whole cell layer by a contraction mechanism. It has been reported that Wnt3a increased the migration rate of myofibroblasts during differentiation and increased fibroblast mediated contraction of Col1 lattices [47]. The fact that these holes are only found on Col1 suggest that Col1 has a unique feature that none of the other tested ECMPs have and one hypothesis is that Col1 provide an anchorage sites for cells movement or contraction.

The *in vitro* differentiation of hES cells towards DE by using a Wnt3a/Activin A protocol resembles the *in vivo* formation of DE. The differentiation with the DE inducing factors Wnt3a and Activin A [5] on the tested ECMP substrates showed similar expression pattern of *POU5F1*, *BRACYURY*, *NANOG*, *SOX17*, *FOXA2* and *CXCR4* as *in vivo* formation of DE [2,48] and previous published *in vitro* protocols [5,7,49] (Fig 5 and S4 Fig). The apparent stronger down regulation of *POU5F1* and up regulation of e.g. *BRACHYURY*, is likely an effect of Col1, which leads to purer cultures of DE cells (Fig 4C). However, gene expression analysis using DNA microarrays showed that *DACT1* was downregulated and *NOS2* was upregulated. It has been shown that *DACT1* inhibits Wnt signalling [39,40], and that *NOS2* activates beta-catenin [41]. This indicates that the effects of Wnt3a could be enhanced by *DACT1* reduction and/or increased *NOS2* activity. This may explain why *BRACHUYRY* showed a large induction the day after Wnt3a stimulation as it has been reported that *BRACHUYRY* is a downstream target of Wnt signalling [50]. The fact that *AXIN2* was not significantly regulated by Col1 indicates that the negative feedback of Wnt signalling by *AXIN2* was not altered in the cells. A possible chain of event could be that Col1 reduces *DACT1* expression and increases *NOS2* expression at the stem cells stage making the cells more susceptible to Wnt3a signalling and thereby increase the fraction of cells entering the differentiation program. Subsequent activation of *DKK1*, which was observed day 6 (Fig 5) a day after *BRACHUERY* expression maximum, may shut down Wnt signalling through a feedback mechanism. *BRACHYURY* is a candidate for activating expression of the *DKK1* gene [51].

The focal adhesions pathway is regulated by binding of ECM to the integrin cell receptors [11]. The focal adhesion pathway affects diverse cellular functions, including cell migration [11] and epithelial to mesenchymal transition (EMT) [52]. *In vivo* cell migration is tightly linked to DE formation during gastrulation where cells undergo EMT [53]. Interestingly, when cancer cells invade tissues or metastasize, they use a mechanism akin to EMT during DE formation and several studies have demonstrated that Col1 can promote EMT in cancerous cells [54–56]. A similar mechanism may be involved in the EMT during DE differentiation of hES cells where Col1 substrate might promotes the EMT and hereby induces the DE differentiation. Is it noteworthy that NOS2, which was upregulated on Col1 substrates according to DNA microarray gene expression data, increase EMT in breast cancer cells [41].

It should be noted that the molecular mechanism of Col1 is currently not known. The effect of Col1 can therefore be biochemical such as presenting other ECMPs or less defined such as affecting cell densities or cell culture rearrangements. Stem cells secrete Fn and Fn binds to Col1 [57] and FN is involved in DE differentiation [18]. We did not observe an additional effect of Fn on DE differentiation when mixed with Col1 (Fig 2) suggesting that Col1 may not positively affect the Fn function. Col1 coating also resulted in a lower initial cell adhesion (2–3 fold) and rapid proliferation compared to other investigated ECMPs (Fig 4). It is therefore possible that the effect of Col1 is due to the initial low cell density. However, Col1 has a similar positive effect on DE differentiation over a large range of obtained cell densities (Fig 2A, green spots), suggesting that the cell density alone is not a dominating parameter. The positive effect of Col1 may be connected to the apparent spotty appearance of cultures on Col1 where stem cells are located in tight clusters (compare culture morphology at day 1, Fig 3). Tight clustering may form micro environment that locally mimic high density cultures. It is noteworthy that cells on Fn+Col2 also appear as clusters, however not as dense as cells on Col1. Fn+Col2 also appears to support efficient DE induction over a large obtained cell range (Fig 2A). However, initial captured cell density (Day 1, Fig 3), clustering of cells (Day 1, Fig 3) or the substrate did not result in cells with different gene expression of key markers at day 4 before Wnt3a induction (Fig 4). This suggests that, at least based on these few investigated genes, the cells at day 4 is similar on the respective ECMP coatings. The positive effect of Col1 may also be linked to the open spaces in the cell culture on Col1 after Wnt3a treatment. Similar open spaces after Wnt3a induction resulted in high percentage of Sox17 positive cells have recently been demonstrated with nanopillars coated with Fn [58]. Significant changes of gene expression of key markers (Fig 5) were observed after Wnt3a induction, which correlates in time with the appearance of open spaces in the cell layer. In conclusion, the observations points to Col1 having effects linked to Wnt3a induction rather than cell density.

ECMPs printed as microarrays on polystyrene slides were used as a screening platform and the protocol resembles previously published protocols [18,44]. We noted that the results from our ECMP microarray screen did not completely correlate with the results obtained in microtitre plates, indicating that the chosen screening platform may not be representative for a corresponding microtitre plate based assays. In the ECMP microarray screen, the ECMPs promoting DE differentiation were associated with low total cell number on the spots. By contrast, the microtitre plate assay resulted in many cases in high total cell numbers while still promoting DE differentiation. This can be one of the reasons why Col1 alone and Col2+Fn are missing as "hits" from the ECMP microarray screen, while performing well in microtitre plate format. Poor adhesion on the ECMP microarray spots of Col1 and Col2+Fn may lead to no cells on the spots that can differentiate day 4. By contrast, cells will always stay in microtitre plates and have long time to adhere. Cell adhesion is poor on Col1 compared to many other ECMPs at day 1 (Fig 4) suggesting that poor adhesion to Col1 is the reason for Col1 not showing up on the ECMP microarray screen. This difference between the ECMP microarray based screen and the microtitre plate can be due to the respective handling of the ECMPs in the case. In the microarray case, the ECMPs were dried on the surface and subsequently treated with ethanol while in the microtitre plate, the ECMPs where kept wet all the time. It can also be due different formats that can cause differences in cell differentiation, i.e. size of culture which is greatly differing between the ECMP microarray experiments and the corresponding microtitre plate well experiments [59].

While the biology of Col1 during development is still unclear, the finding that Col1 promotes DE differentiation has several practical implications. In our study the benchmark coating Fn was above average in the ECMP microarray screen and gave a good cell attachment and a decent DE induction. However, we identified ECMPs which further improve the DE induction, either together with Fn (Col2 and Ne) or alone (Col1). Furthermore, using the semi-optimal protocol with 10ng/ml Activin A on cell seeded on Col1 coating resulted in about 80–90% Sox17 positive cells. Similar fraction of DE cells can be obtained using 100ng/ml Activin A and Fn (S1 Fig). These preliminary results suggest that the right ECMP coating can reduce growth factors usage and hereby the cost, which is important for large scale cell production. In this study we used Col1 from rat tail, since recombinant human Col1 was not available when the study was initiated. However, Col1, like many other ECM proteins, are conserved across species and the rat tail Col1 used can be substituted with recombinant human Col1 [60]. Col1 from rat tail is however, inexpensive and may provide a robust and better alternative to Fn.

## Conclusion

This study identified ECM coatings that induced the differentiation of hES cells into DE cells. The ECMP microarray screen identified several ECMPs that improved stem cell differentiation into DE cells. The majority of the ECMP combinations functioned also in the microtitre well plate format. Col1, Col2 mixed with Fn and Ne1 mixed with Fn induced the DE differentiation to a higher degree than the positive control cultured on Fn alone. However, only cultures on Col1 showed distinct morphology, proliferated faster, and importantly gave purer DE cultures compared to cultures on Fn. Collectively, the results demonstrate an important role for ECMPs in the differentiation of hES cells. The underlying mechanism for the effects of Col1 and the other ECMPs acting positively on *in vitro* differentiation remains unsolved. In practical terms, substituting Fn with Col1 is an easy to implement procedure to increase the differentiation efficiency of hES into DE cells.

## Supporting Information

S1 Fig Quantitative immunocytochemistry showing the percentage of Sox17 positive cells using DE differentiation protocols with different Activin A concentrations (10 or 100ng/ml Activin A) on Fn substrate. DE differentiation with 10ng/ml Activin A gave 57% Sox17 positive cells whereas using 100ng/ml Activin in the DE differentiation resulted in 82% Sox17 positive cells (n = 3–6, mean ± S.E.M., * indicates statistical significant differences, P<0.05).
(TIF)

S2 Fig Fluorescence microscope images of DE differentiation on Fn coating, stained for Sox17, Oct3/4 and DNA (DAPI). Microscope images were captured with InCell Analyzer and similar images were acquired for all the tested ECMP combinations and subsequently quantified (Scale bar = 200μm).
(TIF)

S3 Fig Cell morphology during DE differentiation on different ECMP substrates in microtitre plates at different time points. Representative immunofluorescence images from 3 independent experiments of cells cultured to different time points during the DE differentiation protocol. The cells were stained for Oct3/4, nucleus (DAPI) and F-actin (Alexa Fluor 488 Phalloidin). The tested ECMP combinations were Fn, Col2+Fn, Col1, Ne+Fn and Vn (scale bar = 200μm).
(TIF)

S4 Fig Gene expression analysis of *CXCR4* during the DE differentiation on the different ECMP coatings. *CXCR4*, which is expressed in the definitive endoderm and mesoderm, but not in visceral endoderm, was upregulated during the differentiation for all ECMPs together. This indicated that the differentiation protocol used on the different ECMP substrates direct the hES towards definitive endoderm and not visceral endoderm. (n = 3, mean ±S.E.M.).
(TIF)

S1 Table Outline of the selected ECMPs used in the microarray screen. The table provides information about the individual ECMPs and the rationale for being included in the ECMP microarray screen with reference to the literature.
(DOCX)

S2 Table Sequence of the qPCR primers used in this study. (DOCX)

S3 Table DE differentiation dataset for the ECMP combinations in microtitre plate format. Results from the ECMP microarray screen were validated in microtitre plate format. Cells were seeded on the different ECMP substrates in microtitre plates and subsequently differentiated towards DE. The samples were analysed using quantitative immunofluorescence with the markers Oct3/4, Sox17 and DNA (DAPI). Percentage of Sox17 positive cells and the cell number for each ECMP combination were used to evaluate the differentiation. Excerpt of the results are graphical displayed in Fig 2. (n = 3–5).
(DOCX)

S4 Table Expression of a subset of genes analysed by a gene expression microarray. The gene expression analysis was performed with cells differentiated on different ECMP substrates and compared to cells differentiated on the control substrate Fn. The subset of genes presented here are markers for DE differentiation and pluripotent maintenance. **a)** The table shows the Benjamini-Hochberg corrected P-values for differential expression. The heat map colours indicates the value of the P-value, where green is low, yellow is medium and red is high. The expression of *FOXA2*, *OCT3/4*, *NANOG and SOX2* in cells differentiated on Col1 substrate significant different compared to cells differentiated on Fn substrate. The expression of the genes was significant different in cells differentiated on Col2+Fn, Ne+Fn and Vn substrates compared to cells differentiated on Fn substrate. **b)** The table shows log2 fold changes of gene expression on the different ECMP substrates compared to on Fn. The heat map colours indicate the value of the log2 fold changes, where red is low, yellow is medium and blue is high. The DE markers (*SOX17*, *FOXA2* and *CXCR4*) were upregulated on Col1 substrate compared to on Fn substrate, whereas the pluripotent markers *OCT3/4*, *NANOG*, *SOX2* were downregulated on Col1 substrate compared to on Fn substrate.
(DOCX)

S5 Table Top 10 up- a) and down-regulated (b) genes in cells DE differentiated on Col1 substrate compared to on Fn substrate, analysed by a gene expression microarray. The gene expression analysis on indicated that the expression of several genes in cells differentiated on Col1 substrates where significant up- or down-regulated compared to in cells differentiated on Fn substrates. BH: Benjamini-Hochberg corrected P-values.
(DOCX)

S6 Table Complete set of gene expression results from the gene expression microarray. Gene expression in cell on Col 1 compared to the gene expression in cells on Fn.
(XLSX)

S7 Table Complete set of gene expression results from the gene expression microarray. Gene expression in cell on Col 2+Fn compared to the gene expression in cells on Fn.
(XLSX)

S8 Table Complete set of gene expression results from the gene expression microarray. Gene expression in cell on Vn compared to the gene expression in cells on Fn.
(XLSX)

S9 Table Complete set of gene expression results from the gene expression microarray. Gene expression in cell on Ne compared to the gene expression in cells on Fn.
(XLSX)

S10 Table Complete set of gene expression results from the gene expression microarray. Gene expression in cell on Fn compared to the gene expression in cells on Fn pooled. This analysis was used to determine bias between different cultures on Fn.
(XLSX)
