# Supplementary figures and images for: Collagen Type I Improves the Differentiation of Human Embryonic Stem Cells towards Definitive Endoderm
Source: PLoS One. 2015 Dec 29;10(12):e0145389. doi: 10.1371/journal.pone.0145389 (PMC4694921; doi:10.1371/journal.pone.0145389)

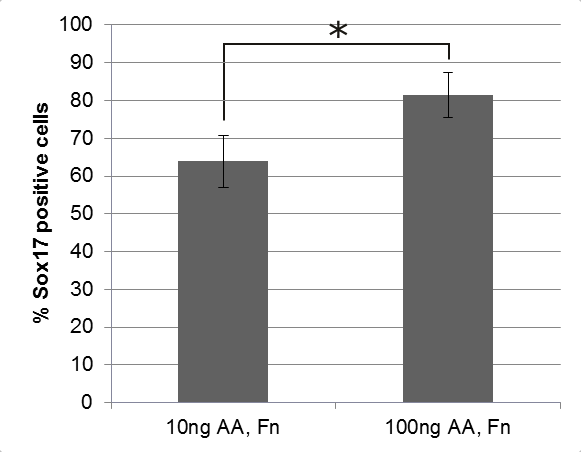

Supplement: S1 Fig — DE differentiation with 10ng/ml Activin A gave 57% Sox17 positive cells whereas using 100ng/ml Activin in the DE differentiation resulted in 82% Sox17 positive cells (n = 3–6, mean ± S.E.M., * indicates statistical significant differences, P<0.05). (TIF) [file pone.0145389.s001.tif]

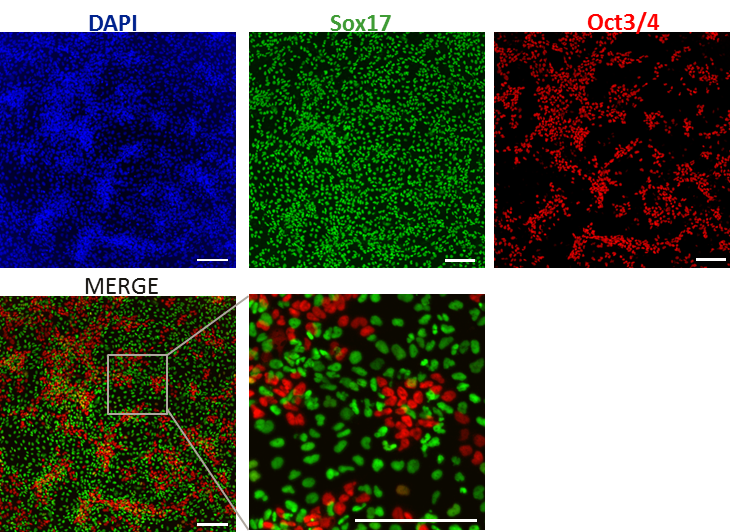

Supplement: S2 Fig — Microscope images were captured with InCell Analyzer and similar images were acquired for all the tested ECMP combinations and subsequently quantified (Scale bar = 200μm). (TIF) [file pone.0145389.s002.tif]

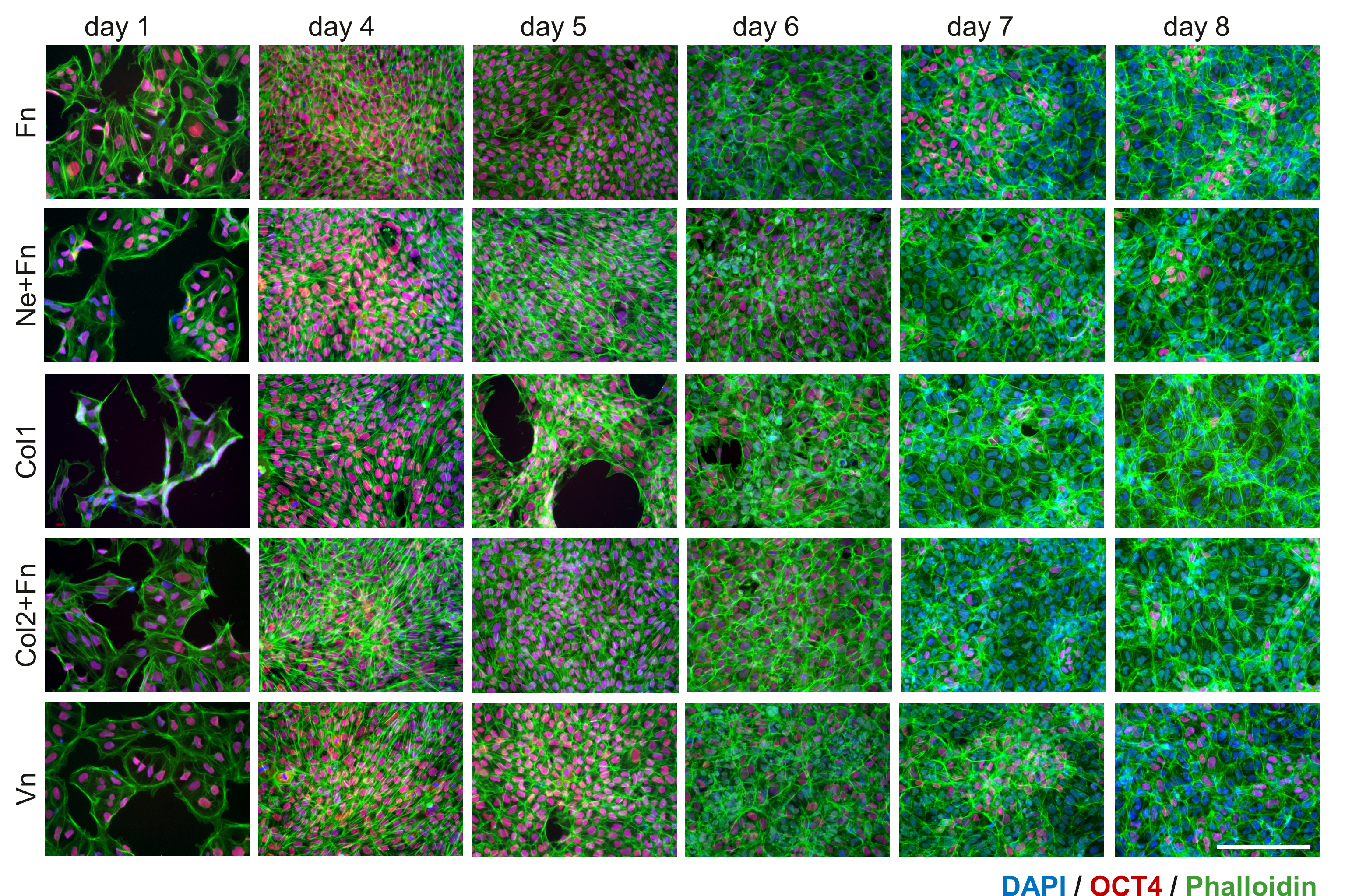

Supplement: S3 Fig — Representative immunofluorescence images from 3 independent experiments of cells cultured to different time points during the DE differentiation protocol. The cells were stained for Oct3/4, nucleus (DAPI) and F-actin (Alexa Fluor 488 Phalloidin). The tested ECMP combinations were Fn, Col2+Fn, Col1, Ne+Fn and Vn (scale bar = 200μm). (TIF) [file pone.0145389.s003.tif]

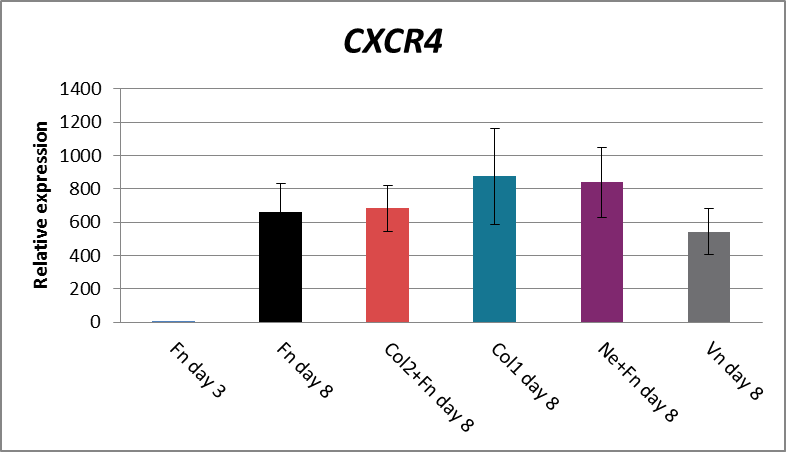

Supplement: S4 Fig — CXCR4, which is expressed in the definitive endoderm and mesoderm, but not in visceral endoderm, was upregulated during the differentiation for all ECMPs together. This indicated that the differentiation protocol used on the different ECMP substrates direct the hES towards definitive endoderm and not visceral endoderm. (n = 3, mean ±S.E.M.). (TIF) [file pone.0145389.s004.tif]
