# Supplementary material for: Collagen Type I Improves the Differentiation of Human Embryonic Stem Cells towards Definitive Endoderm
Source: PLoS One. 2015 Dec 29;10(12):e0145389. doi: 10.1371/journal.pone.0145389 (PMC4694921; doi:10.1371/journal.pone.0145389)
Supplement: S1 Table — The table provides information about the individual ECMPs and the rationale for being included in the ECMP microarray screen with reference to the literature. (DOCX) [file pone.0145389.s005.docx]

| **Protein name** | **Abbreviation** | **Product information** | **Concentration in the array screen**  **1 / 2 / 3 / 4 proteins in a combination^*^** | **Description and rationale for to be included in the array screen** |
| --- | --- | --- | --- | --- |
| Fibronectin | Fn | Sigma, F0895 | 50/ 25/ 16.67/ 12.5 µg/ml | Fn is the current benchmark coating in our laboratory for culturing hES cells. Fn functions as strong cell adhesive substrate where its RGD domain binds to integrins. Previous it has been reported that Fn promote differentiation of hES cells towards definitive endoderm [1]. Another study have showed that Fn is necessary for loss of pluripotency of mouse ES cells [2]. Fn expressed in early stages of development and it has been shown that Fn is essential for gastrulation [3]. |
| Laminin 511 | Ln511 | Biolamina, LN511-02 | 20/10/ 6.67/ 5 µg/ml | Ln511 induce cell renewal of human and mouse ES cells [4,5]. Another have shown that Ln511 induce differentiation of mouse ES cells towards definitive endoderm [6]. Ln511 is expressed by hES cells [7]. |
| Laminin 111 | Ln111 | Biolamina, LN111-02 | 20/10/ 6.67/ 5 µg/ml | Ln511 is expressed in the very early embryonesis [8]. Ln111 is a popular substrate for pluripotent stem cells and have suggested to be involved in epithelial-to-mesenchymal transition in the early transition [9] |
| Laminin 521 | Ln521 | Biolamina, LN521-02 | 20/10/ 6.67/ 5 µg/ml | Ln521 is one of the first ECM proteins expressed in the blastocyst [8]. Studies have shown that Ln521 induce expansion and long term self-renewal of pluripotent stem cells [10] |
| Laminin from human fibroblast | LnH | Sigma, **L4544** | 20/10/ 6.67/ 5 µg/ml | LnH is a mixture of laminins from human plasma (Sigma). Laminins are major component of the ECM, and is expressed in the early embryonic tissue where it contributes to the formation of basal lamina. Laminins are in involved in cell survival, differentiation and migration [11,12]. |
| Vitronectin | Vn | Sigma**, SRP3186** | 50/ 25/ 16.67/ 12.5 µg/ml | Vn functions as adhesive substrate for cell culturing, since it contains integrin binding domain RGD [11]. Vn specific integrins (αV and β5) are up regulated in definitive endoderm derived from hES cells [13]. Further, a study have identified Vn to promote definitive endoderm differentiation of hES cells [1]. |
| Fibronectin adhesions promoting peptide | FnAd | Sigma, **F3667** | 50/ 25/ 16.67/ 12.5 µg/ml | FnAd with the amino acid sequence Trp-Gln-Pro-Pro-Arg-Ala-Arg-Ile, is found the c-terminal heparin-binding domain of fibronectin (Sigma). Previous experience in the laboratory had shown that the hES cells have difficult to adhere to several ECM proteins (unpublished). An adhesive promoting peptide may be beneficial for adhesion of hES cells. FnAD |
| Collagen 1, rat tail | Col1 | Gibco, A10483-01 | 10/ 5/ 3.33/ 2.5 µg/ml | The collagen family is the main components of ECM and function as structural support and binding partners for others ECM proteins [11]. Col1 most abundant ECM protein and is a widely used coating for cell culturing [12]. An array study showed that Col1 had a positive effect on the differentiation of mouse ES cells towards hepatic linages [14]. However, mouse ES cells do not express Col1 as pluripotent or throughout the differentiation into the 3 germ layers [2]. |
| Collagen II | Col2 | Millipore, CC052 | 10/ 5/ 3.33/ 2.5 µg/ml | Col2 add structure and strength to connective tissue and it mainly expressed in cartilage (http://ghr.nlm.nih.gov). |
| Collagen III | Col3 | Millipore, CC054-K | 10/ 5/ 3.33/ 2.5 µg/ml | Col3 is extensively expressed in connecting tissues, including skin, lung, intestinal walls, and the blood vessels walls (http://www.uniprot.org/uniprot/P02461) |
| Collagen IV | Col4 | R&D systems, [3410-010-01](http://www.rndsystems.com/Products/3410-010-01) | 10/ 5/ 3.33/ 2.5 µg/ml | Col4 is the first collagen expressed in the embryo and emerge together with the assembly of the first basal lamina [12]. A study showed that basal lamina from M15 cells contained col4 and it directed mouse ES cells into pancreatic linage [6]. |
| Collagen V | Col5 | Millipore, CC077 | 10/ 5/ 3.33/ 2.5 µg/ml | Col5 is a fibrillar collagen and is a minor component in the all the connective tissue (http://www.uniprot.org/uniprot/P20908) |
| Heparan sulphate proteoglycan | Hep | Sigma, **H4777** | 100/ 50/ 30.33 / 25 ng/ml | Hep is a major compartment of the basal lamina and acts as a reservoir and modulator for various growth factors (FGF, BMP, PDGF, VEGF [15] Hep has been suggested to regulated the differentiation of pancreatic cell lineage [16]. |
| Nidogen-1/Entactin | Nid | R&D Systems, [2570-ND-050](http://www.rndsystems.com/Products/2570-ND) | 30/ 15 / 10 / 7.5 µg/ml | Nid is a primary compartment of the basal lamina and interact with laminin and col4. Laminin-nidogen complex has been shown to be important during organ-development [15]. Nid is expressed by hES cells [7]. |
| Netrin 1 | Ne | R&D Systems, [6419-N1-025](http://www.rndsystems.com/Products/6419-N1) | 5/ 2.5 / 1.67/ 1.25µg/ml | Ne binds to Fn and Col4 and can promote proliferation and migration. Ne is suggested to be involved in neural and pancreatic development and is expressed throughout the nervous system as well as the pancreas [17,18]. |

* The array screen had 1, 2, 3 or 4 proteins within each combination. The concentration for each protein was depended of the number of proteins in the given combination. Here the possible 4 different proteins concentrations is given, when the combination contain 1, 2,3 or 4 proteins.

**References**

1. Brafman D a, Phung C, Kumar N, Willert K. Regulation of endodermal differentiation of human embryonic stem cells through integrin-ECM interactions. Cell Death Differ. Nature Publishing Group; 2013;20: 369–81. doi:10.1038/cdd.2012.138

2. Taylor-Weiner H, Schwarzbauer JE, Engler AJ. Defined extracellular matrix components are necessary for definitive endoderm induction. Stem Cells. 2013;31: 2084–94. doi:10.1002/stem.1453

3. Darribère T, Schwarzbauer JE. Fibronectin matrix composition and organization can regulate cell migration during amphibian development. Mech Dev. 2000;92: 239–250. doi:10.1016/S0925-4773(00)00245-8

4. Rodin S, Domogatskaya A, Ström S, Hansson EM, Chien KR, Inzunza J, et al. Long-term self-renewal of human pluripotent stem cells on human recombinant laminin-511. Nat Biotechnol. Nature Publishing Group; 2010;28: 611–5. doi:10.1038/nbt.1620

5. Domogatskaya A, Rodin S, Boutaud A, Tryggvason K. Laminin-511 but not -332, -111, or -411 enables mouse embryonic stem cell self-renewal in vitro. Stem Cells. 2008;26: 2800–9. doi:10.1634/stemcells.2007-0389

6. Higuchi Y, Shiraki N, Yamane K, Qin Z, Mochitate K, Araki K, et al. Synthesized basement membranes direct the differentiation of mouse embryonic stem cells into pancreatic lineages. J Cell Sci. 2010;123: 2733–42. doi:10.1242/jcs.066886

7. Evseenko D, Schenke-Layland K, Dravid G, Zhu Y, Hao Q-L, Scholes J, et al. Identification of the critical extracellular matrix proteins that promote human embryonic stem cell assembly. Stem Cells Dev. 2009;18: 919–28. doi:10.1089/scd.2008.0293

8. Aumailley M. The laminin family. Cell Adh Migr. 2013;7: 48–55. doi:10.4161/cam.22826

9. Horejs C-M, Serio A, Purvis A, Gormley AJ, Bertazzo S, Poliniewicz A, et al. Biologically-active laminin-111 fragment that modulates the epithelial-to-mesenchymal transition in embryonic stem cells. Proc Natl Acad Sci U S A. 2014;111: 5908–13. doi:10.1073/pnas.1403139111

10. Rodin S, Antonsson L, Niaudet C, Simonson OE, Salmela E, Hansson EM, et al. Clonal culturing of human embryonic stem cells on laminin-521/E-cadherin matrix in defined and xeno-free environment. Nat Commun. 2014;5: 3195. doi:10.1038/ncomms4195

11. Rozario T, DeSimone DW. The extracellular matrix in development and morphogenesis: a dynamic view. Dev Biol. Elsevier Inc.; 2010;341: 126–40. doi:10.1016/j.ydbio.2009.10.026

12. Zagris N. Extracellular matrix in development of the early embryo. Micron. 2001;32: 427–438. doi:10.1016/S0968-4328(00)00011-1

13. Wong JCY, Gao SY, Lees JG, Best MB, Wang R, Tuch BE. Definitive endoderm derived from human embryonic stem cells highly express the integrin receptors alphaV and beta5. Cell Adh Migr. 2010;4: 39–45.

14. Flaim CJ, Chien S, Bhatia SN. An extracellular matrix microarray for probing cellular differentiation. Nat Methods. 2005;2: 119–25. doi:10.1038/nmeth736

15. Kruegel J, Miosge N. Basement membrane components are key players in specialized extracellular matrices. Cell Mol Life Sci. 2010;67: 2879–95. doi:10.1007/s00018-010-0367-x

16. Higuchi Y, Shiraki N, Kume S. In vitro models of pancreatic differentiation using embryonic stem or induced pluripotent stem cells. Congenit Anom (Kyoto). 2011;51: 21–5. doi:10.1111/j.1741-4520.2010.00307.x

17. Lai Wing Sun K, Correia JP, Kennedy TE. Netrins: versatile extracellular cues with diverse functions. Development. 2011;138: 2153–69. doi:10.1242/dev.044529

18. Yebra M, Montgomery AMP, Diaferia GR, Kaido T, Silletti S, Perez B, et al. Recognition of the Neural Chemoattractant Netrin-1 by Integrins α6β4 and α3β1 Regulates Epithelial Cell Adhesion and Migration. 2003;5: 695–707.
