# Supplementary material for: Collagen Type I Improves the Differentiation of Human Embryonic Stem Cells towards Definitive Endoderm
Source: PLoS One. 2015 Dec 29;10(12):e0145389. doi: 10.1371/journal.pone.0145389 (PMC4694921; doi:10.1371/journal.pone.0145389)
Supplement: S2 Table — (DOCX) [file pone.0145389.s006.docx]

| **Target gene** | **Forward primer sequence** | **Reverse primer sequence** |
| --- | --- | --- |
| *TBP* | 5’-CATCACTCCTGCCACGCCAG-3’ | 5’-TGCCGTGGTTCGTGGCTCTC-3’ |
| *GUSP* | 5’-ACGCAGAAAATATGTGGTTGGA-3’ | 5’-GCACTCTCGTCGGTGACTGTT-3’ |
| *NANOG* | 5’-TGAGCTGGTTGCCTCATGTTAT-3’ | 5’-GAAGGAAAAGTATCAAGAAATTGGGATA-3’ |
| *OCT3/4* | 5’-TGGGCTCGAGAAGGATGTG-3’ | 5’-GCATAGTCGCTGCTTGATCG-3’ |
| *BRACHYURY* | 5’-TGCTTCCCTGAGACCCAGTT-3’ | 5’-GATCACTTCTTTCCTTTGCATCAAG-3’ |
| *GOOSECOID* | 5’-GAGGAGAAAGTGGAGGTCTGGTT-3’ | 5’-CTCTGATGAGGACCGCTTCTG-3’ |
| *SOX17* | 5’-GGCGCAGCAGAATCCAGA-3’ | 5’-CCACGACTTGCCCAGCAT-3’ |
| *CXCR4* | 5’-CACCGCATCTGGAGAACCA-3’ | 5’-GCCCATTTCCTCGGTGTAGTT-3’ |
| *FOXA2* | 5’-GGGAGCGGTGAAGATGGA-3’ | 5’-TCATGTTGCTCACGGAGGAGTA-3’ |
| *SOX7* | 5’-ACGCCGAGCTCAGCAAGAT-3 | 5’-TCCACGTACGGCCTCTTCTG-3’ |
| *DKK1* | 5’-ATAGCACCTTGGATGGGTATTCC-3’ | 5’-CTGATGACCGGAGACAAACAG-3’ |
| *AXIN2* | 5’-TACACTCCTTATTGGGCGATCA-3’ | 5’-TTGGCTACTCGTAAAGTTTTGGT-3’ |
