# Supplementary material for: Collagen Type I Improves the Differentiation of Human Embryonic Stem Cells towards Definitive Endoderm
Source: PLoS One. 2015 Dec 29;10(12):e0145389. doi: 10.1371/journal.pone.0145389 (PMC4694921; doi:10.1371/journal.pone.0145389)
Supplement: S3 Table — Results from the ECMP microarray screen were validated in microtitre plate format. Cells were seeded on the different ECMP substrates in microtitre plates and subsequently differentiated towards DE. The samples were analysed using quantitative immunofluorescence with the markers Oct3/4, Sox17 and DNA (DAPI). Percentage of Sox17 positive cells and the cell number for each ECMP combination were used to evaluate the differentiation. Excerpt of the results are graphical displayed in Fig 2. (n = 3–5). (DOCX) [file pone.0145389.s007.docx]

| **ECMP combination** | **%Sox17 positive cells ±SEM** | **Number of cells ±SEM** |
| --- | --- | --- |
| Fn | 63.89 ±2.85 | 20688 ±445 |
| Fn+LnH | 72.81 ±3.23 | 21245 ±283 |
| Ln521+LnH | 72.80 ±3.56 | 17963 ±1995 |
| Ne+Fn | 74.62 ±2.85 | 21782 ±504 |
| FnAd | 0 | 0 |
| Ln511+Fn+Col2+Hep | 57.48 ±8.31 | 20474 ±580 |
| Fn+Ne+Col4 | 63.84 ±7.14 | 20638 ±519 |
| Fn+Ne+Ln511+Col3 | 46.19 ±14.36 | 19729 ±387 |
| Fn+LnH+Hep | 69.17 ±4.55 | 20745 ±92 |
| LnH+Nid+Col1 | 68.38 ±12.82 | 14141 ±3961 |
| Fn+FnAd+Col1 | 76.95 ±1.80 | 20769 ±380 |
| Fn+Vn+LnH | 62.51 ±4.35 | 22025 ±96 |
| Fn+Ne+Ln511 | 56.26 ±8.05 | 18993 ±325 |
| Fn+Ne+Ln511+Col1 | 46.30 ±13.66 | 19400 ±426 |
| Fn+Ne+Col2 | 73.10 ±3.79 | 20237 ±146 |
| Vn+Ne+Ln511+Col4 | 51.13 ±6.10 | 21207 ±954 |
| Fn+Ne+Col5 | 76.56 ±2.66 | 9105 ±3703 |
| Fn+Ne+Col3 | 74.74 ±2.61 | 20552 ±642 |
| Vn+Col1+Nid | 47.17 ±5.74 | 21137 ±1104 |
| Fn+Ne+Nid | 55.41 ±5.51 | 21422 ±840 |
| Vn | 48.36 ±4.44 | 22638 ±782 |
| Fn+Vn | 46.40 ±3.36 | 21803 ±454 |
| Vn+Hep | 51.38 ±4.24 | 23330 ±913 |
| Fn+Vn+Ln511 | 61.19 ±4.35 | 21419 ±104 |
| Fn+Vn+Ln111 | 59.94 ±0.32 | 21555 ±654 |
| Fn+ Vn+Col1 | 56.15 ±1.91 | 21180 ±685 |
| Fn+ Vn+Col3 | 61.24 ±2.67 | 22007 ±994 |
| Fn+Vn+ Ne | 56.63 ±0.97 | 22047 ±864 |
| Fn+Vn+Nid | 52.26 ±3.20 | 21654 ±1025 |
| Fn+Vn+Hep | 42.53 ±7.46 | 22425 ±656 |
| Col2 | 75.42 ±2.89 | 8167 ±1067 |
| Fn+Col2 | 73.13 ±2.56 | 21141 ±379 |
| Col2+Vno | 48.68 ±3.64 | 22436 ±606 |
| Ln511 | 58.61 ±3.03 | 20399 ±491 |
| Fn+Ln511 | 58.62 ±5.97 | 19598 ±791 |
| Col2+Ln511 | 62.90 ±5.73 | 20971 ±233 |
| Ne | 0 | 0 |
| Col2+Ne | 64.33* | 2257 |
| Vn+Ne | 47.02 ±3.61 | 22903 ±770 |
| Nid | 55.96 ±11.32 | 13776 ±6001 |
| Fn+Nid | 56.51 ±6.63 | 20852 ±675 |
| Col2+Nid | 53.83 ±6.87 | 22319 ±77 |
| Vn+Nid | 48.54 ±2.52 | 22575 ±757 |
| Ln511+Nid | 58.89 ±7.65 | 21027 ±977 |
| Ln511+Ne | 62.94 ±6.15 | 17311 ±800 |
| Ne+Nid | 0 | 0 |
| FnAd+Col1 | 79.67 ±3.37 | 15404 ±1583 |
| Fn+Col2+Vn+Ln511+Ne+Nid | 53.77 ±5.57 | 20723 ±508 |
| Col2+Nid | 0 | 0 |
| Ln511+Vn | 46.48 ±7.65 | 20445 ±852 |
| Ne+LnH | 0 | 0 |
| Vn+Hep | 50.91 ±1.91 | 22531 ± 581 |
| LnH+Col1 | 78.94 ±6.47 | 17324 ±851 |
| LnH+Col1+Ne | 77.41 ±7.00 | 14562 ±2199 |
| Col1 | 84.30 ±1.21 | 19834 ±425 |
| LnH | 0 | 0 |
| Col1+Fn | 76.11 ±4.90 | 23413.11 ±179 |
| Vn+LnH | 41.66 ±6.81 | 21716 ±1085 |
| Col1+Nid | 60.72 ±6.85 | 20250 ±2286 |
| Vn+Col1 | 50.41 ±6.38 | 23277 ±854 |
| LnH+Nid | 49.83* | 1946 |
| Col1+Ne | 80.63 ±0.89 | 10284 ±3122 |
| FnAD+ Fn | 67.40 ±6.06 | 21694 ±613 |
| Fn+Ne+Col1+Col2+LnH | 81.38 ±3.37 | 17593 ±2243 |
| BSA | 0 | 0 |

*only one experiment with cells and SEM cannot be calculated.
