# Supplementary material for: Collagen Type I Improves the Differentiation of Human Embryonic Stem Cells towards Definitive Endoderm
Source: PLoS One. 2015 Dec 29;10(12):e0145389. doi: 10.1371/journal.pone.0145389 (PMC4694921; doi:10.1371/journal.pone.0145389)
Supplement: S4 Table — The gene expression analysis was performed with cells differentiated on different ECMP substrates and compared to cells differentiated on the control substrate Fn. The subset of genes presented here are markers for DE differentiation and pluripotent maintenance. a) The table shows the Benjamini-Hochberg corrected P-values for differential expression. The heat map colours indicates the value of the P-value, where green is low, yellow is medium and red is high. The expression of FOXA2, OCT3/4, NANOG and SOX2 in cells differentiated on Col1 substrate significant different compared to cells differentiated on Fn substrate. The expression of the genes was significant different in cells differentiated on Col2+Fn, Ne+Fn and Vn substrates compared to cells differentiated on Fn substrate. b) The table shows log2 fold changes of gene expression on the different ECMP substrates compared to on Fn. The heat map colours indicate the value of the log2 fold changes, where red is low, yellow is medium and blue is high. The DE markers (SOX17, FOXA2 and CXCR4) were upregulated on Col1 substrate compared to on Fn substrate, whereas the pluripotent markers OCT3/4, NANOG, SOX2 were downregulated on Col1 substrate compared to on Fn substrate. (DOCX) [file pone.0145389.s008.docx]

**a)**

| **Benjamini-Hochberg corrected p-values for differential expression** | | | | | | |  |
| --- | --- | --- | --- | --- | --- | --- | --- |
| **ECMPs compared to fibronectin controls** | **p_*SOX17*** | **p_*FOXA2*** | **p_*GOOSECOID*** | **p_*CXCR4*** | **p_*OCT3/4*** | **p_*NANOG*** | **p_*SOX2*** |
| **Collagen 1** | 0.06204 | 0.002 | 0.54771 | 0.08676 | 0.00014 | 0.00051 | 0.00024 |
| **Collagen 2/Fibronectin** | 0.34044 | 0.30394 | 0.92176 | 0.46658 | 0.23717 | 0.61211 | 0.25118 |
| **Netrin 1/Fibronectin** | 0.43262 | 0.39634 | 0.77715 | 0.49071 | 0.37685 | 0.78037 | 0.37066 |
| **Vitronectin** | 0.60035 | 0.99941 | 0.89369 | 0.86855 | 0.99647 | 0.99748 | 0.82301 |

**
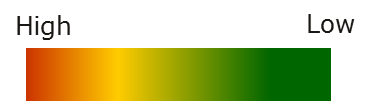
**

**b)**

| **log2 fold changes** | |  |  |  |  |  |  |
| --- | --- | --- | --- | --- | --- | --- | --- |
| **ECMPs condition compared to fibronectin controls** | ***SOX17*** | ***FOXA2*** | ***GOOSECOID*** | ***CXCR4*** | ***OCT3/4*** | ***NANOG*** | **SOX2** |
| **Collagen 1** | 0.22976 | 0.55119 | -0.2401 | 0.27587 | -1.4698 | -1.3011 | -1.3335 |
| **Collagen 2/Fibronectin** | 0.19152 | 0.2855 | 0.07404 | 0.18384 | -0.5811 | -0.2514 | -0.5788 |
| **Netrin 1/Fibronectin** | 0.23748 | 0.36081 | 0.24586 | 0.26358 | -0.5957 | -0.2162 | -0.6337 |
| **Vitronectin** | -0.2026 | -0.0004 | -0.2 | -0.1104 | -0.0084 | 0.0053 | 0.22742 |

**
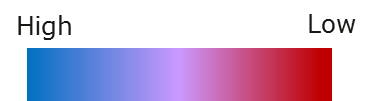
**
