# Supplementary material for: Collagen Type I Improves the Differentiation of Human Embryonic Stem Cells towards Definitive Endoderm
Source: PLoS One. 2015 Dec 29;10(12):e0145389. doi: 10.1371/journal.pone.0145389 (PMC4694921; doi:10.1371/journal.pone.0145389)
Supplement: S5 Table — The gene expression analysis on indicated that the expression of several genes in cells differentiated on Col1 substrates where significant up- or down-regulated compared to in cells differentiated on Fn substrates. BH: Benjamini-Hochberg corrected P-values. (DOCX) [file pone.0145389.s009.docx]

**a)**

| **Ensembl_gene_ID** | **Gene_**  **name** | **Unipro**  **Acc .no.** | **log2 Fold Change** | **Average Expression** | **p-Value** | **BH-adjusted p-Value** | **Bonferroni corrected p-value** |
| --- | --- | --- | --- | --- | --- | --- | --- |
| ENSG00000165617 | DACT1 | Q9NYF0 | -1,67 | 10,12 | 3,98E-06 | 0,000913489 | 0,163514443 |
| ENSG00000204531 | POU5F1 | Q01860 | -1,47 | 13,86 | 1,30E-08 | 0,000135832 | 0,000535369 |
| ENSG00000114315 | HES1 | Q14469 | -1,45 | 10,24 | 2,82E-06 | 0,000816302 | 0,115914834 |
| ENSG00000212993 | POU5F1B | Q06416 | -1,40 | 14,07 | 2,27E-08 | 0,000135832 | 0,000933646 |
| ENSG00000119900 | OGFRL1 | Q5TC84 | -1,36 | 11,05 | 8,48E-06 | 0,001299423 | 0,348514842 |
| ENSG00000181449 | SOX2 | P48431 | -1,33 | 11,77 | 9,16E-08 | 0,000235054 | 0,00376306 |
| ENSG00000168234 | TTC39C | Q8N584 | -1,33 | 10,05 | 3,79E-08 | 0,000179526 | 0,001555387 |
| ENSG00000165617 | DACT2 | Q9NYF1 | -1,32 | 13,28 | 1,80E-08 | 0,000246066 | 0,013072824 |
| ENSG00000204531 | POU5F1 | Q01860 | -1,31 | 13,28 | -8,81E-08 | 0,000240158 | 0,011311032 |
| ENSG00000135046 | ANXA2 | P04084 | -1,27 | 13,27 | -1,04E-06 | 0,000186985 | -0,004545092 |

**b)**

| **Ensembl_gene_ID** | **Gene_**  **name** | **Unipro**  **Acc .no.** | **log2 Fold Change** | **Average Expression** | **p-Value** | **BH-adjusted p-Value** | **Bonferroni corrected p-value** |
| --- | --- | --- | --- | --- | --- | --- | --- |
| ENSG00000007171 | NOS2 | P35228 | 1,38 | 8,06 | 3,03E-07 | 0,000355449 | 0,012440725 |
| ENSG00000257017 | HP | P00738 | 1,35 | 10,54 | 5,98E-08 | 0,000224592 | 0,002457488 |
| ENSG00000261701 | HPR | P00739 | 1,35 | 10,54 | 5,98E-08 | 0,000224592 | 0,002457488 |
| ENSG00000163638 | ADAMTS9 | Q9P2N4 | 1,30 | 9,50 | 2,09E-08 | 0,000135832 | 0,000857241 |
| ENSG00000198336 | MYL4 | P12829 | 1,23 | 13,43 | 4,65E-07 | 0,000410303 | 0,019099892 |
| ENSG00000180209 | MYLPF | Q96A32 | 1,16 | 11,25 | 7,46E-08 | 0,000235054 | 0,003065709 |
| ENSG00000175206 | NPPA | P01160 | 1,10 | 8,19 | 1,06E-06 | 0,000512119 | 0,043530106 |
| ENSG00000144810 | COL8A1 | P27658 | 1,08 | 9,35 | 1,41E-07 | 0,000259916 | 0,005804759 |
| ENSG00000198336 | MYL4 | P12829 | 1,06 | 10,79 | 4,21E-07 | 0,000402716 | 0,017296376 |
| ENSG00000106631 | MYL7 | Q01449 | 1,03 | 14,57 | 6,04E-09 | 0,000124046 | 0,000248091 |
